# Effect of *Lonomia obliqua* Venom on Human Neutrophils

**DOI:** 10.3390/toxins13120908

**Published:** 2021-12-18

**Authors:** João Alfredo Moraes, Genilson Rodrigues, Daniel Guimarães-Bastos, Vany Nascimento-Silva, Erik Svensjö, Mariana Renovato-Martins, Markus Berger, Jorge Guimarães, Christina Barja-Fidalgo

**Affiliations:** 1 Laboratório de Biologia RedOx, Instituto de Ciências Biomédicas, Universidade Federal do Rio de Janeiro, Ave. Carlos Chagas Filho 373, Prédio Novo do ICB, Sala 3 3 Andar, Rio de Janeiro 21941-902, Brazil; daniel.guimaraes.bastos@gmail.com (D.G.-B.); barja-fidalgo@uerj.br (C.B.-F.); 2 Laboratório de Farmacologia Celular e Molecular, Universidade do Estado do Rio de Janeiro, Rio de Janeiro 20551-030, Brazil; biologogenilson@gmail.com (G.R.); vanysilva66@gmail.com (V.N.-S.); 3 Instituto de Biofísica Carlos Chagas Filho, Universidade Federal do Estado do Rio de Janeiro, Rio de Janeiro 21941-902, Brazil; erik.svensjo@gmail.com; 4 Laborotário de Imunologia e Metabolismo, Universidade Federal Fluminense, Niterói 22410-201, Brazil; marianarenovato@id.uff.br; 5 Laboratório de Bioquímica Farmacológica, Centro de Pesquisa Experimental, Hospital de Clínicas de Porto Alegre, Porto Alegre 90035-003, Brazil; markusoliveira@hcpa.edu.br (M.B.); jguimaraes14@gmail.com (J.G.); 6 Centro de Biotecnologia, Universidade Federal do Rio Grande do Sul, Porto Alegre 91501-970, Brazil

**Keywords:** Lonomia, venom, neutrophil, reactive oxygen species

## Abstract

The significant incidence of deforestation in South America culminates in the contact of humans with typical forests species. Among these species, one may highlight *Lonomia obliqua* caterpillar, which, when touched by humans, can poison them through their bristles. Therefore, better acknowledging the mechanisms involved in envenomation caused by *Lonomia obliqua* caterpillar bristle extract (LOCBE) may contribute to further treatments. Recently, we demonstrated that LOCBE induces a pro-inflammatory profile in endothelial cells; thus, we decided to investigate the effects of LOCBE on human polymorphonuclear neutrophils (PMN), which are the first leukocytes that migrate to the inflammatory focus. Our results showed that treatment with LOCBE induced PMN chemotaxis together with alterations in actin cytoskeleton and focal adhesion kinase (FAK) activation, favoring migration. Concurrently, LOCBE induced PMN adhesion to matrix proteins, such as collagen IV, fibronectin, and fibrinogen. Moreover, we observed that LOCBE attenuated PMN apoptosis and increased reactive oxygen species (ROS) production together with nuclear factor kB (NF-κB) activation—a redox-sensitive transcription factor—as well as interleukin (IL)-1β and IL-8 release. We call attention to the ROS-dependent effect of LOCBE on increased cell migration once an antioxidant treatment reverted it. In summary, we report that LOCBE activates PMN, inducing pro-inflammatory responses modulated by ROS.

## 1. Introduction

Lonomism is the envenomation promoted by caterpillars from the Lonomia genus. There are two species of Lonomia involved in human accidents: *Lonomia obliqua* and *Lonomia Achelous* [1]. Envenomation caused by *Lonomia achelous* dates from 1967 in Venezuela [2], and this caterpillar can be found in Venezuela, Bolivia, Colombia, Ecuador, French Guyana, Suriname [3,4], and north of Brazil. *Lonomia obliqua* is found mainly in the south of Brazil, and recently the species appears to be spreading to the southeast of Brazil [5], contributing to the increase in reported accidents [6]. Beyond Brazil, *Lonomia obliqua* is present in Argentina, Paraguay, and Uruguay [1]. The deforestation process aggravates the prevalence of accidents with the caterpillar, which occurs when the victim touches the caterpillars, leading to contact with the caterpillar’s bristles [7].

Envenomation caused by human contact with the caterpillar *Lonomia obliqua* triggers a hemorrhagic clinical profile installed within a period of up to 72 h [8,9,10,11]. In parallel to blood coagulation and the intense fibrinolytic activity, proteases and other active enzymes present in *Lonomia obliqua* caterpillar bristle extract (LOCBE) may also cause direct effects on vascular cells, establishing a vascular inflammation process [12,13].

When a tissue injury occurs, the first step to stabilize an inflammation is the endothelial cell’s recognition of a danger signal, which in turn will induce local vasodilatation and expose adhesion molecules to induce neutrophil (PMN) rolling, adhesion, and, finally, PMN transmigration to the injured tissue. PMN migration occurs according to a gradient concentration of chemotactic agents, and PMNs have their half-lives prolonged to ensure that they can solve the possible threat. Different signaling pathways mediate these processes through reactive oxygen species (ROS), generated during PMN activation [14].

Recently we showed that LOCBE activates the endothelium, promoting leukocyte rolling and adhesion [13]. The main objective of the present study was to evaluate the effects in vitro of LOCBE on PMN activation and functionality.

## 2. Results

### 2.1. In Vivo Effect of LOCBE on Plasma Leakage and Leukocyte Accumulation

**Figure 1:**
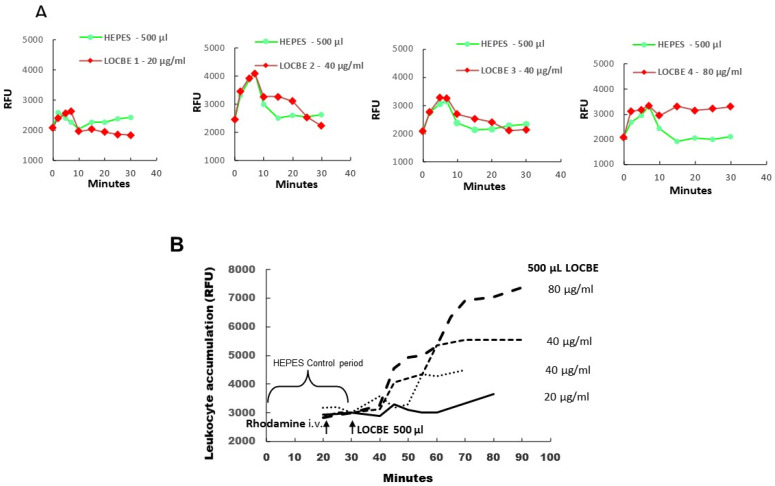
All hamsters (*n* = 4) received an intravenous (i.v.) injection of FITC-dextran 150 kDa before LOCBE venom or HEPES buffer application. Rhodamine (100 μg/kg) was given i.v. 10 min before LOCBE venom application (500 μL of 20, 40, or 80 μg/mL). (**A**) Topical application of 500 μL of HEPES buffer at 0 min (Green curves) and 30 min later of 500 μL of LOCBE venom 20, 40, or 80 μg/mL (red curves) during 10 min of interrupted superfusion. LOCBE venom 20 μg/mL caused no increase in plasma leakage (RFU = relative fluorescent units) and 40 μg/mL (*n* = 2) caused a slight increase and 80 μg/mL resulted in a prolonged and irreversible increase in plasma leakage until the end of the experiment (90 min). (**B**) Topical LOCBE venom application in 4 different HCPs caused dose-related increments of leukocyte accumulation, reaching maximal values between 60 and 90 min after LOCBE venom application.

Firstly, we applied LOCBE to a hamster cheek pouch to demonstrate microvascular alterations induced by the venom. In this essay, we tried to mimic in vivo an envenomation condition, and therefore we topically applied three different concentrations of LOCBE (20–80 μg/mL) on the exposed hamster cheek pouch (HCP). Thus, we observed that the HCP treated with LOCBE 20 μg/mL for 10 min showed little increase in plasma leakage (RFU = relative fluorescent units) but more with 40 μg/mL, that became persistent with the highest dose of the venom, 80 μg/mL (Figure 1A and Supplemental Figure S1A,B). We observed a pronounced increase in leukocyte accumulation in the postcapillary and larger venules that were dose-dependent (Figure 1B and Supplemental Figure S1C,D).

As it is well established that PMN is the first defensive line that migrates to an inflammatory focus, we aimed to evaluate the role of this cell in poisoning promoted by LOCBE.

### 2.2. Effect of LOCBE on PMN Migration

**Figure 2:**
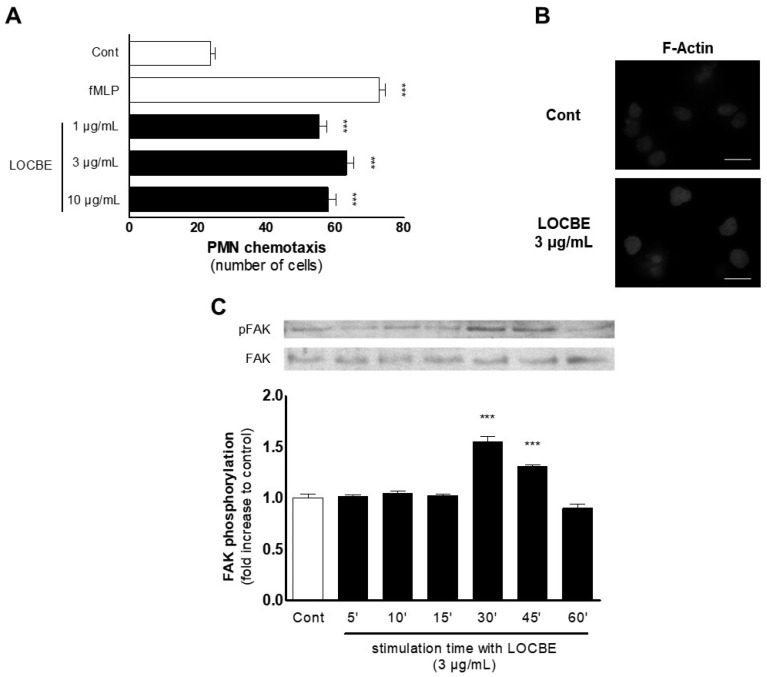
LOBCE presents chemotactic activity. (**A**) PMNs were inserted in the upper compartment of a 48-well Boyden chamber (5 × 10^4^ cells per well) to test their migration towards LOBCE (at 1, 3, and 10 μg/mL), inserted in the lower compartment of the chamber, separated by a 5 μm pore-sized polyvinylpyrrolidone-free polycarbonate membrane. The peptide fMLP (100 nM) was used as a positive control. *** *p* < 0.001 compared to the control group (*n* = 3). (**B**) PMNs (10^6^ cells per well) were incubated in the presence or absence of LOBCE (3 μg/mL) for 30 min. Then, PMNs were fixed, incubated for two hours with phalloidin-tetramethylrhodamine B isothiocyanate, and examined in a microscope equipped for epifluorescence. Scale bar: 10 μm. (**C**) PMNs (10^6^ cells per well) were incubated in the presence or absence of LOBCE (3 μg/mL) for varying times (up to 60 min). Then, PMN were lysed, and extracts were run through SDS-PAGE with acrylamide gels, transferred to polyvinylidene fluoride membranes, blocked with BSA 5%, and probed with anti-pFAK or anti-actin. Immunoreactive bands were visualized using an ECL solution through a ChemiDoc Imaging System. *** *p* < 0.001 compared to the control group (*n* = 3).

To evaluate the effect of LOCBE on human PMN, we studied the migratory effect for one hour in a modified Boyden chamber. LOCBE showed to be a chemotactic agent in all concentrations tested (1–10 μg/mL), similar to fMLP (100 nM) stimulus (Figure 2A). LOCBE effect on PMN migration was observed as a classic bell shape, so we chose the concentration of 3 mg/mL for further experiments.

To assess the mechanisms involved in the LOCBE effect, we evaluated the cytoskeleton dynamics through phalloidin-rhodamine analysis by fluorescence microscopy after 30 min of treatment. As expected, LOCBE (3 mg/mL) induced several cytoskeleton rearrangements compared to the control group, inducing high levels of F-actin (Figure 2B). Proceeding with the investigation about the underlying mechanisms involved in the LOCBE effect on PMN migration, we performed a molecular approach, investigating the focal adhesion kinase (FAK), a pivotal molecule to cell migration. Once activated, FAK forms the focal adhesion, which allows cell movement. The focal adhesion formation occurs because FAK promotes actin cytoskeleton rearrangement via adapter molecules [15]. Thus, we observed by Western blotting that LOCBE (3 μg/mL) was able to promote FAK activation after 30 min (up to 45 min) of treatment (Figure 2C).

### 2.3. Effect of LOCBE on PMN Adhesion

**Figure 3:**
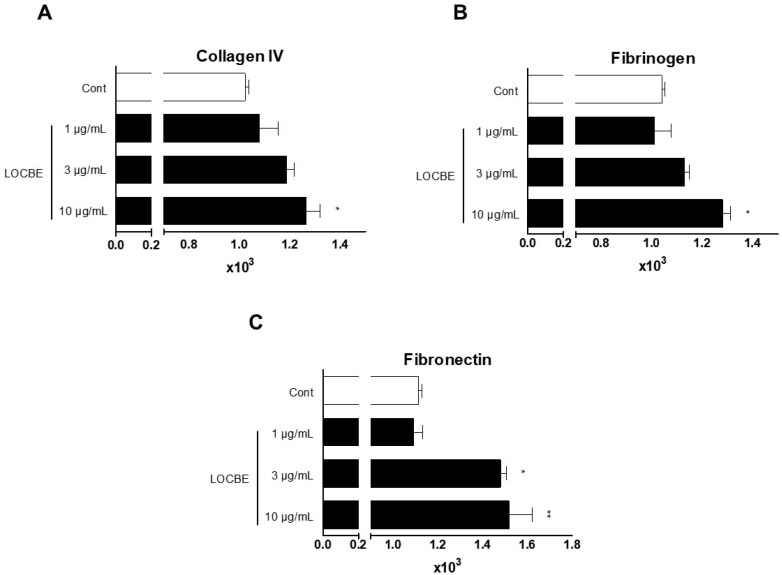
LOBCE stimulates PMN adhesion to matrix proteins. PMNs (5 × 10^4^ cells per well) were incubated with CMFDA (10 μM) for 30 min. Then, PMNs were washed, incubated in the presence or absence of LOBCE (1–10 μg/mL), and seeded in a 96-well black plate which was previously coated with (**A**) collagen IV, (**B**) fibrinogen, or (**C**) fibronectin for 30 min at 37 °C in a 5% CO_2_ air atmosphere. After this time, the plate was washed, and the adherent PMNs were measured through CMFDA fluorescence at excitation and emission wavelengths of 492 and 517 nm, respectively. * *p* < 0.05 compared to the control group, ** *p* < 0.01, compared to the control group (*n* = 3).

It is well established that focal adhesion is crucial for cellular adhesion, and that pro-inflammatory stimuli can induce cluster and integrin activation, which can also facilitate cell adhesion [16]. Once we observed that LOCBE induced FAK activation, we wondered about the adhesion function of PMN treated with the venom. So, we tested PMN adhesion in different matrix proteins, which bind to different adhesion molecules (integrins). We showed that LOCBE could induce PMN adhesion to collagen IV, fibronectin, and fibrinogen (Figure 3).

### 2.4. Effect of LOCBE on PMN ROS Production

**Figure 4:**
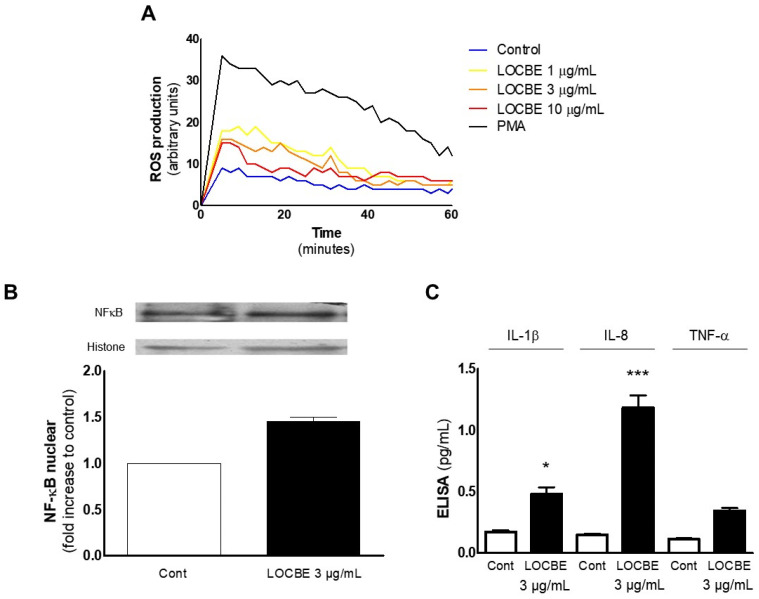
LOBCE induces ROS-dependent NF-κB activation in PMN. (**A**) PMNs (5 × 10^4^ cells per well) were incubated in the presence or absence of LOCBE (1–10 μg/mL) for one hour. Then, PMNs were washed, incubated with luminol (100 μM), and total ROS production was measured by chemiluminescence emitted from luminol oxidation (*n* = 3). (**B**) PMNs (10^6^ cells per well) were incubated in the presence or absence of LOCBE (3 μg/mL) for 1.5 h. Then, PMNs were lysed, and extracts were run through SDS-PAGE with acrylamide gels, transferred to polyvinylidene fluoride membranes, and probed with anti-NF-κB or anti-histone. Immunoreactive bands were visualized using an ECL solution through a ChemiDoc Imaging System (*n* = 3). (**C**) PMNs (10^6^ cells) were incubated in the presence or absence of LOCBE (3 μg/mL) for three hours. Then, PMNs were centrifuged, the supernatant was collected and submitted to quantification of IL-1β, IL8, and TNF-α using a sandwich ELISA kit according to the manufacturer’s instructions (*n* = 3), * *p* < 0.05, *** *p* < 0.001.

Another essential function of PMNs is the ability to generate ROS, which can be applied to an oxidative burst or to activate ROS-dependent signaling pathways [17]. Initially, we observed that LOCBE could induce ROS production in all concentrations tested (Figure 4A). Next, we investigated the activation of ROS-dependent signaling pathways. Nuclear factor kB (NF-kB) is a ROS-dependent molecule, which can translocate to the nucleus and induce several pro-inflammatory genes, such as interleukin (IL)-1β, IL-8, and TNF-α [18,19]. In agreement with this idea, we observed that LOCBE induced nuclear translocation of NF-kB after 30 min (Figure 4B) and production of IL-1b and IL-8 after three hours (Figure 4C).

### 2.5. Effect of LOCBE on PMN Survival

**Figure 5:**
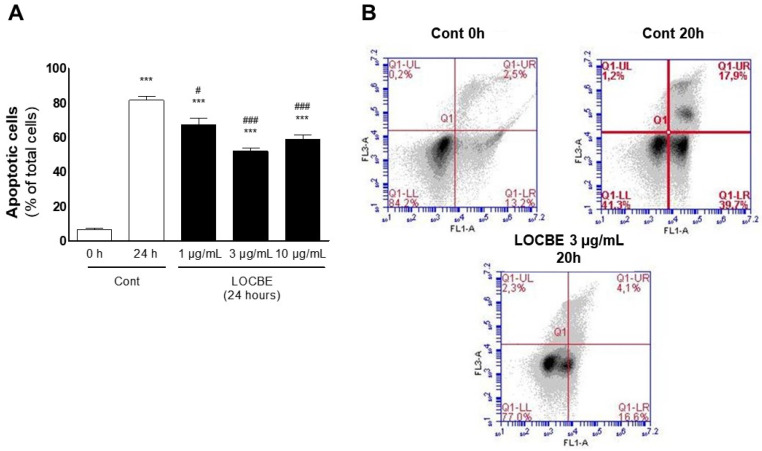
LOBCE induces apoptosis protection in PMN. PMN (10^6^ cells) were incubated in the presence or absence of LOCBE (1–10 μg/mL) for 20 h. Then, PMNs were centrifuged, and (**A**) stained with Diff-Quick™ staining kit, and apoptotic cells were counted using optical microscopy. The bars represent the average of three independent experiments with SEM. Asterisks indicate statistical significance *** *p* < 0.001 compared to control group at 0 h; # *p* < 0.05, compared to control group after 24 h, ### *p* < 0.001 compared to control group after 24 h (*n* = 3); or (**B**) labeled with annexin V-FITC and PI to be analyzed using flow cytometry. Annexin V+ cells (apoptotic cells) were read at the FL1 channel, and PI+ cells (necrotic cells) were read at the FL3 channel (*n* = 3).

PMNs must migrate to the inflammatory site in acute inflammation events. During migration, PMNs become activated, and their half-lives are prolonged [20]. Therefore, we asked about the LOCBE effect on PMN survival. We observed, using optical microscopy, that after 20 h of LOCBE treatment, the number of cells presenting morphological features of apoptosis (i.e., condensed nucleus, cell volume shrinkage) diminished (Figure 5A). To confirm this effect, we also observed through cytometry that the number of annexin-V positive cells decreased after LOCBE (3 μg/mL) treatment (Figure 5B).

### 2.6. Oxidative Effect of LOCBE on PMN

**Figure 6:**
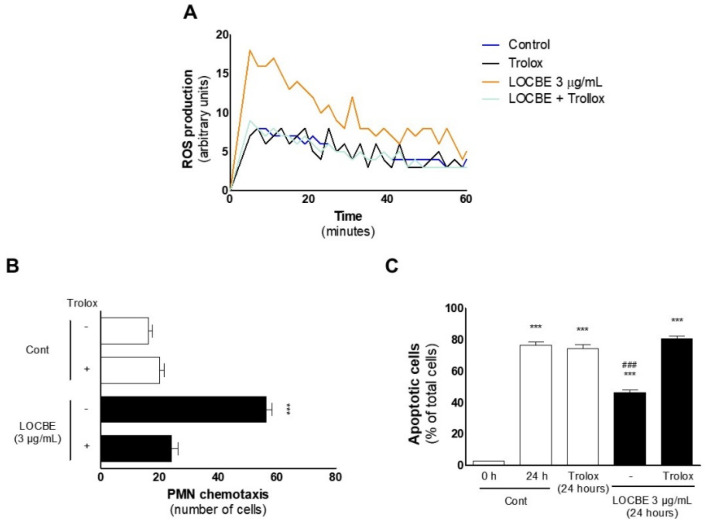
LOBCE-induced migration and apoptosis protection are ROS-dependent. (**A**) PMNs (5 × 10^4^ cells per well) were incubated in the presence or absence of LOCBE (3 μg/mL) for one h. Then, PMNs were washed, incubated with luminol (100 μM), and total ROS production was measured by chemiluminescence emitted from luminol oxidation (*n* = 3). (**B**) PMNs were pre-incubated in the presence or absence of Trolox™ (100 μM) for 5 min before insertion in the upper compartment of a 48-well Boyden chamber (5 × 10^4^ cells per well), and LOBCE (3 μg/mL) was inserted in the lower compartment. DMEM medium alone was used as a negative control. PMNs migrated through a 5 μm pore-sized polyvinylpyrrolidone-free polycarbonate membrane. *** *p* < 0.001 compared to LOBCE group alone. (*n* = 3) (**C**) PMNs (10^6^ cells) were pre-incubated in the presence or absence of Trolox™ (100 μM) for 5 min. Then, PMNs were treated or not with LOCBE (3 μg/mL) for 20 h. After this time, PMN were centrifuged and stained with Diff-Quick™ staining kit, and apoptotic cells were counted using optical microscopy. *** *p* < 0.001 compared to the control group at 0 h, ^###^ *p* < 0.001 compared to the LOBCE 3 μg/mL + Trolox group (*n* = 3).

After identifying that LOCBE can induce ROS production, we performed this assay in the presence of the anti-oxidant Trolox™, and we observed that it could inhibit LOCBE’s (3 μg/mL) effect (Figure 6A). So, we asked about ROS importance in the two crucial functions of activated PMN mentioned before: migration and survival. To this end, we performed these assays again, treating PMNs concomitantly with Trolox™ (100 mM). Both effects were inhibited, suggesting that they are mediated by ROS (Figure 6B,C).

## 3. Discussion

Previously, we showed that LOCBE induces vascular inflammation, triggering a robust pro-inflammatory profile in endothelial cells [13]. Herein, we focused on evaluating the LOCBE effect in the pivotal agent of the acute inflammatory response: PMNs.

This is the first time that the *L. obliqua* venom’s effects on PMNs are described. The caterpillar venom triggers an intense local inflammatory response upon contact with the caterpillar’s bristle, so we believe that the venom can modulate PMN function, playing a significant role in initiation and amplification phases of inflammation. In previous studies by our group, it was shown that LOCBE is able to trigger cell migration, proliferation, and production of proinflammatory cytokines in endothelial and aortic smooth muscle vascular cells [11,12,13]. We also demonstrated previously that *L. obliqua* venom has a toxin able to generate active kallikrein from prekallikrein in rat plasma. Intravascular generated kallikrein during the envenomation can induce ROS production in the kidney (thus contributing to kidney injury), and also can increase ROS and migration of smooth muscle vascular cells. All these events seem to be dependent on kallikrein generation triggered by the venom in plasma, since the pre-treatment of envenomed rats with aprotinin (a kallikrein inhibitor) was able to decrease ROS generation in vascular cells and protect renal tissue from injury [11]. Previous proteomic studies also identified some class of molecules, such as lipocalins and hemolins, which can be involved in a potential cytoprotective effect [21]. It is well established that purified hemolins are able to induce fibroblasts proliferation and protect these cells from apoptosis [22].

To understand the envenomation promoted by LOCBE, we applied a high venom concentration directly in the hamster cheek pouch to simulate the contact of large amounts of the venom. So, we observed that LOCBE could induce plasma leakage and leukocyte accumulation, as presented in Supplementary Figure S1A,B that shows an image of plasma leakage, and Supplementary Figure S1C,D that shows leukocyte accumulation after treatment with LOCBE 80 μg/mL. Once we observed in vivo that LOCBE induced plasma leakage with the highest dose of the LOCBE venom with a pronounced leukocyte accumulation in venules, followed by migration into extravascular tissue, we evaluated the LOCBE effect on PMNs in vitro. Given that both the concentrations of LOCBE within the circulation or the concentrations of PMNs exposed to this are unknown, we tested smaller amounts of LOCBE in PMNs. Thus, we observed that LOCBE at 3 μg/mL induced PMN activation, modulating different functions. We characterize LOCBE as a potent chemotactic agent to PMNs, an inductor of ROS generation, and an anti-apoptotic modulator in PMNs. Taking these effects together, we can affirm that LOCBE is a pro-inflammatory agent to PMNs. On the other hand, Waismam et al. [23] showed that a peptide extracted from LOCBE (Lopap) had no effects on PMN recruitment to the microvasculature. We believe that the observed effects in this work can help us better understand the mechanisms involved in the envenomation process, such as FAK activation, ROS production, and NF-kB activation.

It is well established that cellular migration is a process dependent on a well-operationally actin cytoskeleton [24]. Once PMN migration and actin cytoskeleton rearrangement were identified, we sought to investigate the mechanisms involved in these effects. Thus, we investigated the FAK involvement, a key molecule in cell migration. Activated FAK forms the focal adhesion, which allows cell movement [16]. In this study, we showed that LOCBE (3 μg/mL) could promote FAK activation while inducing PMN adhesion to fibronectin, fibrinogen, and collagen IV. We hypothesized that LOCBE can activate α1b1 and α5b1 integrins, facilitating PMN adhesion to fibronectin, fibrinogen, and collagen IV.

Large amounts of ROS have been reported to be related to cell damage, while lower amounts to cell signaling [25]. Among the redox-sensitive molecules, NF-kB is crucial to PMN survival (apoptosis inhibition), and, once activated, translocates to the nucleus, promoting gene transcription. Similarly, NF-kB activation was detected 30 min after PMN stimulation with LOCBE following the time observed for ROS production. Corroborating these data, we also observed that LOCBE could induce IL-1β and IL-8 release, a known NF-kB target gene [18,19]. Our data agree with Pinto et al. [6], which showed that LOCBE strongly induced IL-8 expression in fibroblasts through gene expression analysis. After six hours of treatment, we also observed that LOCBE (3–10 μg/mL) was able to induce COX-2 expression (Supplementary Figure S2), suggesting that the pro-inflammatory profile observed in this work could be established via COX-2 activation.

After characterizing LOCBE as a pro-oxidant molecule, we investigated the role of ROS in LOCBE effects and showed that the antioxidant Trolox™ inhibited LOCBE-induced migration and cell survival. These data show that LOCBE effects on PMNs are modulated by ROS, since the concomitant use of an antioxidant abolished them.

In addition to PMNs, recent papers described the LOCBE effect on platelets [4,8] and endothelial cells [13,26] through different mechanisms. These papers have all shown the role of LOCBE inducing platelet aggregation, erythrocyte lysis, and a pro-inflammatory profile in endothelial cells.

## 4. Conclusions

**Figure 7:**
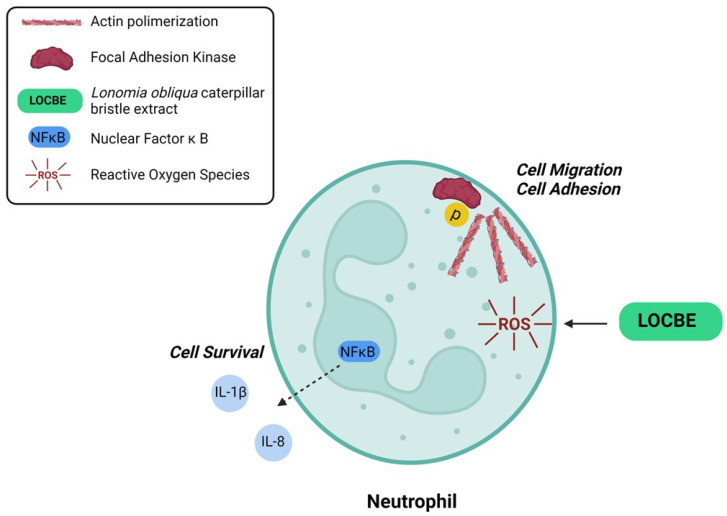
Proposed model for LOBCE effect on PMNs. LOCBE induces PMN migration, survival, triggers ROS production, and activates NF-κB, leading to release of IL-1β and IL-8.

This work showed that LOCBE acts on PMN migration and survival. Furthermore, we demonstrated that LOCBE triggers ROS production and activates NF-κB, a redox-sensitive pathway (Figure 7). Finally, we pointed out the possibility of using antioxidants in the treatment of envenomation promoted by LOCBE.

## 5. Material and Methods

### 5.1. Reagents

HEPES, trypsin, EDTA, bovine serum albumin (BSA), PMSF, benzamidine, leupeptin, and soybean trypsin inhibitor were from Sigma-Aldrich (St. Louis, MO, USA). TroloxTM was from Calbiochem (Darmstadt, Germany). Dulbecco’s Modified Eagle’s Medium (DMEM) and fetal calf serum (FCS) were from GIBCO-BRL (Carlsbad, CA, USA). Antibodies anti-actin, anti-COX-2, anti-pFAK, and anti-NF-κB were purchased from Santa Cruz Biotechnology (Santa Cruz, CA, USA). ECL system was obtained from Pierce Biotechnology (Rockford, IL, USA). LOCBE was obtained as previously described [25].

### 5.2. Lonomia obliqua Caterpillar Bristle Extract (LOCBE)

LOCBE was obtained as previously described [27]. *L. obliqua* caterpillars were collected in endemic areas from the Brazilian states of Rio Grande do Sul and Santa Catarina, and kindly donated by the Centro de Informações Toxicológicas (CIT), Porto Alegre, Rio Grande do Sul, Brazil. The venomous secretion was obtained by cutting bristles, macerating them in cold phosphate-buffered saline (PBS) (pH 7.4), and centrifuging at 9600× *g* for 20 min at 4 °C. The supernatant (*L. obliqua* caterpillar bristle extract—LOCBE) was collected and maintained at −80 °C until use. The protein concentrations of the LOCBE samples were determined using a BCA (bicinchoninic acid) assay kit (Pierce, Rockford, IL, USA). The total number of caterpillars used for bristle extract preparation was 30 specimens, and the protein concentration of the LOCBE samples was 2.10 mg/mL. The total amount of venom extracted per caterpillar was 2.8 mg. All caterpillars were at sixth instar and 4–5 cm in size. LOCBE doses used in this work were based on previous studies by our group, in which venom effects were tested on endothelial and vascular smooth muscle cells in culture [11,12,13].

### 5.3. Intravital Microscopy

Hamsters were purchased from Anilab (São Paulo, Brazil) and maintained in our animal facilities. The experiments were conducted with male hamsters, three months old, weighing 110–120 g. Altogether, four hamsters were used, and the procedures were approved by the local ethical committee (IBCCF, license protocol number 014). Animals were anesthetized by injection of sodium pentobarbital (intraperitoneal) supplemented with α-chloralose (2.5% *w*/*v*, solution in saline, intravenous—i.v.) through a femoral vein catheter, as previously described [28,29]. During the experimental procedures, anesthesia was monitored by reflex measurement and supplemented with α-chloralose (2.5% *w*/*v*, solution in saline, i.v.) through a femoral vein catheter whenever required. A tracheal cannula (PE 190) was inserted to facilitate spontaneous breathing, and the body temperature was maintained at 37 °C by a heating pad monitored with a rectal thermistor. The hamster cheek pouch (HCP) was prepared for intravital microscopy (IVM) as reported [28,30]. The microcirculation of the HCP was observed after intravenous injection of the tracer FITC-dextran 150 kDa (100 mg/kg), and leukocyte accumulation was observed after intravenous injection of the tracer rhodamine 123 using an Axioskop 40 microscope, objective 4× and oculars 10× (Carl Zeiss, Oberkochen, Germany), equipped with an appropriate filter (490/520 nm, FITC-dextran, and 524/580 nm, rhodamine) for observations of fluorescence in epiluminescence (Colibri 2, Carl Zeiss, Oberkochen, Germany). HCPs were continuously superfused with an HEPES-bicarbonate-buffered saline solution (pH 7.4; composition in mM: 110.0 NaCl, 4.7 KCl, 2.0 CaCl_2_, 1.2 MgSO_4_, 18.0 NaHCO_3_, 15.39 HEPES, and 14.61 Na HEPES) at a constant rate of 5 mL/min. A digital camera, AxioCamHRc, and a computer with the AxioVision 4.4 software program (Carl Zeiss) were used for image analysis of arteriolar diameter and total fluorescence in a representative rectangular area (5 mm^2^) of the HCP. After an initial 30 min control period of continuous superfusion to verify normal microvascular flow in all vessels and an absence of plasma leakage, HCPs were subjected to topical applications of different combinations of treatments during interrupted superfusion in 500 μL of HEPES buffer on top of the HCP tissue during interrupted superfusion that was re-established after 10 min. The experiments were performed by capturing images with 5 min intervals of a representative area (5 mm^2^) of the HCP before HEPES buffer or LOCBE (20–80 μg/mL) application for 10 min, and, subsequently, until the end of the experiment (50–90 min).

### 5.4. PMN Isolation

PMNs were isolated from healthy volunteers (approval #38257914.7.0000.5259 from the Ethics Committee Hospital Universitário Pedro Ernesto, UERJ, Rio de Janeiro, Brazil) in the presence of 5% EDTA, using a discontinuous Percoll gradient. Volunteers provided informed consent before blood collection at all times. The study was carried out following the ethical standards established in the Declaration of Helsinki of 1975 and its later amendments. After isolation, PMNs were suspended in DMEM serum-free and submitted to the assays, except in the case of apoptosis assay, in which PMNs were suspended in DMEM containing 10% FBS.

### 5.5. Cell Migration Assay

Aliquots of 10^6^ cells/mL were seeded in a 48-well modified Boyden chamber with 5 μm pore-sized polyvinylpyrrolidone-free polycarbonate membranes (Neuroprobe, Inc., Gaithersburg, MD, USA), as previously described [31]. Some samples were pre-incubated with Trolox™ for 5 min before addition to the upper chamber, in a total volume of 50 μL (5 × 10^4^ cells) per well. LOCBE (1–10 μg/mL) or fMLP (100 nM) were added to the lower chamber (in a total volume of 27 μL). After incubation at 37 °C in a 5% CO_2_ air atmosphere for one hour, the upper side of the membrane was scrapped, and cells that had migrated to the lower membrane surface were fixed, stained by Giemsa, and counted by optical microscopy (400× magnification).

### 5.6. Cytochemistry

PMNs (10^6^ cells/mL) were incubated in the presence or absence of LOCBE for 30 min at 37 °C in a 5% CO_2_ air atmosphere. After incubation, the cells were cytocentrifuged and mounted in coverslips to be fixed with a PBS solution containing 5% paraformaldehyde. Then, PMNs were permeabilized with Triton (0.2%) for 5 min, washed three times with PBS, and incubated for two hours with phalloidin-tetramethylrhodamine B isothiocyanate (1:1000) at room temperature. Coverslips were mounted on a slide using a PBS solution containing 20 mM N-propyl gallate and 80% glycerol before examination under a microscope (model BX40 Olympus, Tokyo, Japan) equipped for epifluorescence. The images were analyzed using Photoshop software (Adobe Systems, San Jose, CA, USA).

### 5.7. Western Blot Analysis

PMNs (10^6^ cells/mL) were incubated in the presence or absence of LOCBE at 37 °C in a 5% CO_2_ air atmosphere for 15 min (FAK), 1.5 h (NF-kB), or 4 h (COX). Then, PMNs were lysed with a buffer containing 50 mM HEPES, pH 6.4, 1 mM MgCl_2_, 10 mM EDTA, 1% Triton X-100, 1 μg/mL DNase, 0.5 μg/mL Rnase, 1 mM PMSF, 1 mM benzamidine, 1 μg/mL leupeptin, and 1 μM/mL soybean trypsin inhibitor.

Total protein content was quantified, and extracts were boiled at 100 °C for 5 min. Equal amounts of protein (50 μg) were loaded into each lane and separated using sodium dodecyl sulfate-polyacrylamide gel electrophoresis (SDS-PAGE) with acrylamide gels. Following the transfer, the blots were probed with antibodies against anti-actin, anti-COX-2, anti-pFAK, or anti-NF-κB overnight. On the next day, membranes were washed and probed with appropriate HPR-labeled secondary antibodies, and after extensive washings, membranes were incubated with ECL solution to visualize immunoreactive bands using ChemiDoc Imaging System (Bio-Rad, Hercules, CA, USA).

### 5.8. Cell Adhesion Assay

PMNs (10^6^ cells/mL) were incubated for 30 min with CMFDA (10 μM). After incubation, PMNs were washed, treated or not with LOCBE, and seeded in a 96-well black plate coated with BSA, collagen IV, fibronectin, or fibrinogen for 30 min at 37 °C in a 5% CO_2_ air atmosphere. After incubation, the plate was washed, and the adherent PMNs were measured through CMFDA fluorescence, at excitation and emission wavelengths of 492 and 517 nm, respectively, which were analyzed using Envision™ Multilabel Plate Reader (PerkinElmer, Waltham, MA, USA).

### 5.9. ROS Production Assay

PMNs (10^6^ cells/mL) were treated with LOCBE for one hour at 37 °C in a 5% CO_2_ air atmosphere. Following treatment, cells were centrifuged, seeded in 96-well white plates (5 × 10^4^ cells/well), incubated with luminol (100 μM), and total ROS production was measured by chemiluminescence emitted from luminol oxidation, using Envision™ Multilabel Plate Reader.

### 5.10. ELISA

PMNs (10^6^ cells) were incubated in the presence or absence of LOCBE for three hours at 37 °C in a 5% CO_2_ air atmosphere. After centrifugation, the supernatant was collected and submitted to quantification of IL-1β, IL8, and TNF-α. According to the manufacturer’s instructions, these proteins were quantified using a sandwich ELISA kit (Peprotech, Cranbury, NJ, USA).

### 5.11. Apoptosis Analysis

PMNs (10^6^ cells/mL) were suspended in DMEM with 10% FBS and pre-incubated in the presence or absence of Trolox™ (100 μM) for 5 min at 37 °C in a 5% CO_2_ air atmosphere. Then, PMNs were treated with LOCBE for 20 h. After treatment, PMNs were centrifuged and submitted to two different protocols:

Morphologically: PMNs were stained with Diff-Quick™ staining kit (Panotico-Laborclin, Pinhais, Parana, Brazil), and apoptotic cells were counted using optical microscopy; annexin V binding: PMNs were fixed and labeled with annexin V-FITC (Abcam, Cambridge, United Kingdom) and propidium iodide (PI) to be analyzed using flow cytometry (Accuri C6TM, Ann Arbor, MI, USA). Annexin V^+^ cells (apoptotic cells) were read at the FL1 channel, and PI^+^ cells (necrotic cells) were read at the FL3 channel.

### 5.12. Statistical Analysis

Statistical significance was assessed by ANOVA, followed by Bonferroni’s *t*-test, and a *p* < 0.05 was taken as statistically significant.

## Data Availability

The data presented in this study are available on request from the corresponding author.

## Supplementary Materials

The following are available online at https://www.mdpi.com/article/10.3390/toxins13120908/s1, Figure S1: Intravital microscopy images showing plasma leakage and leukocyte accumulation in the hamster cheek pouch. Figure S2: LOCBE induces COX-2 expression.
