# Supplementary material for: Effect of Lonomia obliqua Venom on Human Neutrophils
Source: Toxins (Basel). 2021 Dec 18;13(12):908. doi: 10.3390/toxins13120908 (PMC8707409; doi:10.3390/toxins13120908)
Supplement: Supplementary file 1 [file toxins-13-00908-s001.zip › toxins-1512447.pdf]

Supplementary Materials: Effect of *Lonomia obliqua* Venom on Human Neutrophils

João A. Moraes, Genilson Rodrigues, Daniel Guimarães-Bastos, Vany Nascimento-Silva, Erik Svensjö, Mariana Renovato-Martins, Markus Berger, Jorge Guimarães and Christina Barja-Fidalgo

SUPPLEMENTARY RESULTS

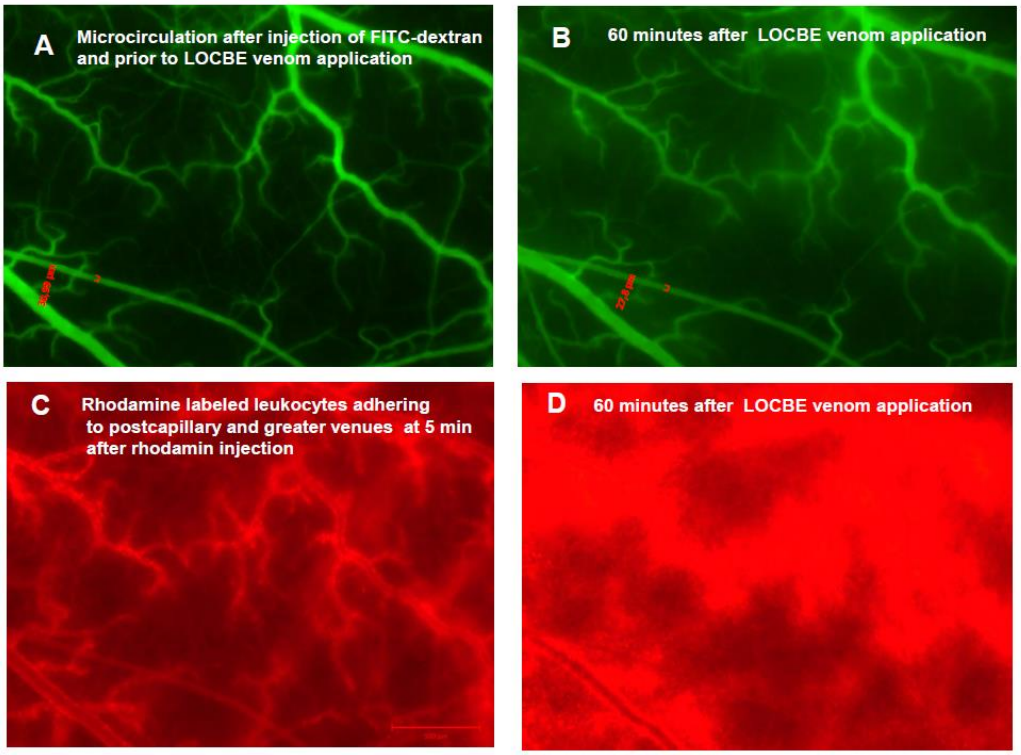

Supplementary Figure S1: Intravital microscopy (IVM) images of the hamster cheek pouch (HCP) microcirculation before and after local application of LOCBE venom solution. A. IVM image of HCP microcirculation before HEPES buffer and LOCBE venom application. B. Same área at 60 min after LOCBE venom application 80 µg/ml showing diffuse extravasation of FITC-dextran around postcapillary venules. C. IVM image at 5 min after rhodamine i.v. injection showing adherence of rhodamine-labeled leukocytes to endothelium in postcapillary and larger venules. D. Same área 60 min after LOCBE venom 80 µg/ml application showing much-increased adherence in venules and accumulation of migrated leukocytes in extravascular areas (https://zenodo.org, access date: 1 December 2021).

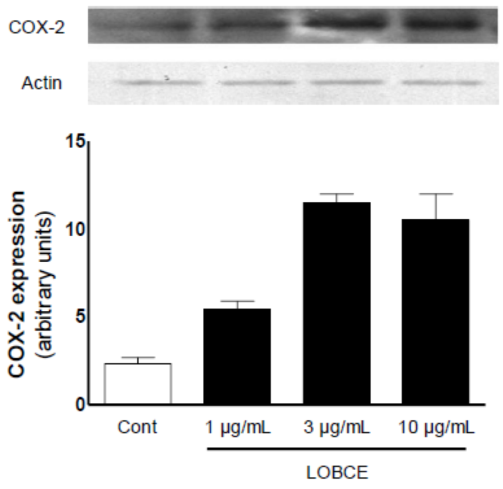

Supplementary Figure S2.:. LOCBE induces COX-2 expression. PMN (106 cells per well) were incubated in the presence or absence of LOCBE (1-10 µg/mL) for 6 h. Then, PMN were lysed, and extracts were run through SDS-PAGE with acrylamide gels, transferred to polyvinylidene fluoride membranes, and probed with anti-COX-2 or anti-Actin. Immunoreactive bands were visualized using an ECL solution through a ChemiDoc Imaging System (n = 3) (https://zenodo.org, access date: 1 December 2021).
